# Adult use of health services: A cross-sectional population-based study, Pelotas, Brazil, 2012 and 2021

**DOI:** 10.1590/S2237-96222026v35e20250460.en

**Published:** 2026-04-20

**Authors:** Larissa Adna Neves Silva, Felipe Mendes Delpino, Lilian Munhoz Figueiredo, Elaine Thumé, Elaine Tomasi, Luiz Augusto Facchini, Bruno Pereira Nunes

**Affiliations:** ¹ Universidade Federal de Pelotas, Programa de Pós-Graduação em epidemiologia, Pelotas, RS, Brazil; ² Universidade Federal de Pelotas, Programa de Pós-Graduação em Odontologia, Pelotas, RS, Brazil; ³ Universidade Federal de Pelotas, Pós-Graduação em Enfermagem, Pelotas. RS, Brazil

**Keywords:** Health Services, Health Inequalities, COVID-19, Adults, Cross-Sectional Studies., Servicios de Salud, Inequidades en Salud, COVID-19, Adultos, Estudios Transversales.

## Abstract

**Objectives:**

To compare prevalence of health service use among adults in the urban area of ​​Pelotas in 2012 and 2021 and to assess socioeconomic inequalities in those years.

**Methods:**

This was a cross-sectional, population-based study of adults living in the urban area of ​​Pelotas in 2012 and 2021. Unadjusted and adjusted analyses were performed using Poisson regression to assess health service use in the 30 days prior to the surveys, based on sex, age, education, race/skin color, economic class, marital status and self-rated health. The slope index of inequality was used to measure inequalities.

**Results:**

The sample consisted of 2,925 participants in 2012 and 5,717 in 2021. Prevalence of health service use increased from 29.3% (95% confidence interval - 95%CI 27.6; 31.1) to 34.4% (95%CI 33.2; 35.6). Among health service users, use of Brazilian Unified Health System facilities increased from 45.7% (95%CI 42.7; 48.7) to 69.8% (95%CI 67.8; 71.7), with a proportional reduction in the use of private services. In 2021, greater dependence on public services was found among women (63.2%), poorer individuals (31.4%) and younger individuals (56.7%). There was an increase in inequalities between economic classes.

**Conclusion:**

There was an increase in the use of health services and greater reliance on public services in 2021, especially among socially vulnerable groups. Socioeconomic inequalities widened, reflecting changes in access patterns in the context of the economic and health crises.

## Resumen

**Objetivos:**

Comparar la prevalencia del uso de servicios de salud entre adultos del área urbana de Pelotas en 2012 y 2021, y evaluar las desigualdades socioeconómicas en estos años.

**Métodos:**

Estudio poblacional transversal de adultos residentes en el área urbana de Pelotas en 2012 y 2021. Se realizaron análisis crudos y ajustados mediante regresión de Poisson para evaluar el uso de servicios de salud en los 30 días previos a las encuestas, con base en sexo, edad, educación, raza/color de piel, clase económica, estado civil y salud percibida. Se utilizó el índice de desigualdad absoluta para medir las desigualdades.

**Resultados:**

La muestra estuvo compuesta por 2.925 participantes en 2012 y 5.717 en 2021. La prevalencia del uso de servicios de salud aumentó del 29,3% (intervalo de confianza del 95% - IC95% 27,6; 31,1) al 34,4% (IC95% 33,2; 35,6). Entre los usuarios, el uso de unidades del Sistema Único de Salud aumentó del 45,7% (IC95% 42,7; 48,7) al 69,8% (IC95% 67,8; 71,7), con una reducción proporcional en el uso de servicios privados. En 2021, se observó una mayor dependencia de los servicios públicos entre las mujeres (63,2%), las personas más pobres (31,4%) y las personas más jóvenes (56,7%). Hubo un aumento de las desigualdades por clase económica.

**Conclusión:**

En 2021, se observó un aumento en el uso de los servicios de salud y una mayor dependencia de los servicios públicos, especialmente entre los grupos socialmente vulnerables. Las desigualdades socioeconómicas se acentuaron, lo que refleja los cambios en los patrones de acceso en el contexto de la crisis económica y sanitaria.

## Introduction 

Universal health systems play a crucial role in reducing social inequalities and ensuring access to health services. Access refers to the relationship between a person’s demand for health services and their entry into the health system and depends on perceived need, socioeconomic characteristics and service availability ^1^.

Despite challenges since its creation in the 1990s, the Brazilian Unified Health System (*Sistema Único de Saúde*, SUS) has improved health equity throughout Brazil, as it has increased access to health services through the expansion of Primary Health Care by means of the Family Health Strategy. However, since 2016, with the implementation of fiscal austerity policies ^2^ and the onset and course of the coronavirus pandemic, these achievements have been negatively impacted, with constant threats to the provision of services and guaranteed access to health care ^2^ ^,^ ^3^. 

Between 2020 and 2021, in high-income countries such as the United States, Canada, the United Kingdom and Australia, there were significant reductions in outpatient visits for routine checkups, disease screening and preventive care, particularly at the beginning of the COVID-19 pandemic ^4^. In Brazil, during 2020, significant reductions were seen in the availability of medical and dental appointments and in procedures involving prenatal care and care for people with diabetes ^3^ ^,^ ^5^. During this period, approximately one in four individuals did not seek care for a health problem or missed a checkup ^6^.

In 2020, Pelotas, a city with approximately 350,000 inhabitants, 62% of the population was covered by family health teams - a 28 percentage point increase compared to 2012, when coverage was only 34% ^7^. Between 2012 and 2021, the municipality underwent significant changes in its primary care structure, including team reorganizations and redirection of services during the pandemic, with a direct impact on the availability of services and the population’s access to them ^7^ ^-^ ^10^. Expanding coverage did not eliminate the inequalities in access to health services that have been widely described ^10^ ^-^ ^12^ and became intensified during the pandemic ^13^.

Given that delays in care and lack of access can have short- and long-term consequences for the population’s health, it is important to assess changes in access to services. The objectives of this article were to compare the prevalence of health service use among adults in the urban area of ​​Pelotas, a city in the state of Rio Grande do Sul, in 2012 and 2021, and to assess socioeconomic inequalities in those years.

## Methods

Design

This was a cross-sectional study that used data from two cross-sectional population-based surveys carried out in 2012 and 2021 in the urban area of ​​Pelotas, a city in the state of Rio Grande do Sul.

Setting

The first study was conducted during a master’s degree consortium arranged by the *Universidade Federal de Pelotas* Epidemiology Postgraduate Program in 2012 ^14^. Within the consortium, each master’s degree student calculated the sample size based on the specific outcome to be investigated, incorporating 10% increases for possible losses and refusals and 15% to control for confounding factors and to adjust for the design effect. The largest estimated sample was 3,120 adults and 800 adolescents. In order to achieve this number, it was estimated that 1,560 households in the municipality would need to be included. In order to account for the effect of the sampling design, 130 census tracts were randomly selected, with visits to 12 households per tract ^15^.

In 2012, the municipality had 495 census tracts, according to the 2010 Demographic Census conducted by the Brazilian Institute of Geography and Statistics. Due to the unavailability of information on the socioeconomic status of the tracts, they were ordered according to their original numbering. Based on this ordering, systematic sampling was conducted. Each tract had information on the total number of households, identified by a starting number and an ending number, totaling 107,152 households in the municipality ^15^.

This total was divided by the 130 census tracts to be selected, thus defining the systematic range of 824 households. Census tract selection began with the random number 634, drawn using Stata, and was carried out systematically, respecting the probability proportional to the number of households in each tract. In each selected household, all individuals aged 10 or older were invited to participate in the study.

A total of 3,671 interviews were conducted, with 13.4% losses and refusals. In cases of refusal, the households were not replaced with others. Interviews not conducted after three attempts on different days and times, with one attempt being made by a study supervisor, were considered losses and refusals. Further details of the sampling process can be found in other publications ^15^.

The second survey took place in 2021, as part of the cohort project entitled 

“Emergency Department Use and Artificial Intelligence in Pelotas” (EAI PELOTAS). That study used baseline data collected between September and December 2021. The sampling process was also conducted in multiple stages. The first consisted of the random selection of 100 census tracts based on estimates updated by the Brazilian Institute of Geography and Statistics in 2019. Subsequently, a proportional number of households was defined for each census tract. Finally, one resident aged 18 or older was interviewed in each household; if more than one eligible resident was selected, a random number generator application ^16^ made the selection. Further details about the survey can be found in another publication ^16^ and on the study website: https://wp.ufpel.edu.br/eaipelotas/.

Participants

In the 2012 survey, all eligible residents (aged 20 or older) of each selected household were included in the analysis, whereas in 2021 only one resident per household was interviewed and included in the analysis.

 Variables and measurement 

The outcome was health service use, defined as the proportion of individuals who sought care and received it. In both studies, the question used was “Since (a given day in the last month), have you been treated at any health service?” Thus, the reference period corresponded to the 30 days prior to the interview.

The selected independent variables were: sex (male, female), age in complete years (20-29, 30-39, 40-49, 50-59, 60+), race/skin color (White, Black, Brown [Brazilian mixed race], Asian, Indigenous), education in complete years (up to 4, 5-8, 9-11, 12+), marital status (no partner, has a partner), economic classification as per the Brazilian Association for Population Studies (*Associação Brasileira de Empresas de Pesquisa*, ABEP ^17^ (A - wealthiest and B, C, D and E - poorest, and categorized as A/B, C, D/E) and self-reported health (excellent/great/very good, good, fair, poor/very poor). The economic classification, age and education variables were used to estimate inequalities, as described in the Statistical Methods subsection.

Self-reported health status was assessed by means of the question “How do you rate your health?” in both surveys. Economic classification, as per the ABEP 2008 version, was based on ownership of certain goods and services (such as color television, radio, bathroom, automobile, washing machine, DVD player, refrigerator and freezer), having a domestic employee paid on a monthly basis, and the level of education of the head of the household. Each item had a specific score, which were added together to provide an overall final score. Categorization was performed according to the cutoff points indicated in the ABEP economic classification criteria document ^17^.

The following variables were used in order to characterize the last time healthcare was received in the month prior to the interview: legal nature of the health service (SUS, private/health insurance) and type of care provision service (Primary health care center, Emergency department/hospital, Private practice/doctor’s office, other). The “other” category corresponded to services related to the city’s medical specialty center, psychosocial care center, and health services in other cities. 

Statistical methods 

Descriptive analyses were performed, calculating prevalence and respective 95% confidence intervals (95%CI). Differences in prevalence were assessed using the heterogeneity test and, when applicable, the trend test. After describing the bivariate analyses, adjusted prevalence rates and their respective 95%CI were calculated using a Poisson regression model, with mutual adjustment between variables. All variables were added simultaneously. Adjusted estimates were obtained through post-estimation, which enabled the predicted probabilities of the dependent variable for each category of independent variables to be calculated, keeping the other variables constant. 

P-values>0.05 were considered statistically significant. All analyses were performed using Stata software, version 18.0, and took into account the complex sampling design through the pre-defined sampling plan.

In order to assess inequalities, we used the Slope Index of Inequality, calculated through logistic regression for ordinal categorical variables such as age, education and economic status. The index expressed the adjusted absolute difference in the probability of using health services between the lowest and highest values ​​of the variable analyzed-such as younger individuals compared to older individuals, or those with less education compared to those with more education-considering the entire distribution of intermediate groups. When the value was zero, that indicated no inequality. Positive values ​​indicated greater use of services among older groups, those with more education, or those with better socioeconomic conditions. Negative values ​​indicated greater use among younger individuals, those with less education or those in more disadvantaged social situations ^18^.

Double stratification was also performed, that is, the analyses considered two variables simultaneously: the legal nature of the health services and other independent variables, such as education level, age and economic status. For example, the study analyzed whether the use of public or private services varied between groups with different levels of education, age groups or economic classes. 

Sensitivity analyses were performed to assess the robustness and consistency of the main findings. These analyses included: i) reanalysis of the 2012 data, considering only one resident per household, to standardize the unit of analysis and enable comparability with the 2021 data, which were collected based on a single individual per household; and ii) evaluation of the 2021 data without applying the post-stratification strategy, to examine the impact of weighting on correcting potential selection biases arising from the sampling process.

## Results 

In 2012 and 2021, 2,925 and 5,717 adults and elderly individuals were assessed, respectively. In both years, just over half of the studied population was female (58.9% in 2012 and 54.2% in 2021), the majority identified as White (80.1% in 2012 and 77.5% in 2021), had between 9 and 11 years of schooling (28.0% in 2012 and 36.1% in 2021) and reported good self-rated health (42.0% in 2012 and 57.5% in 2021). Most participants were in the 20-59 age group (77.2% in 2012 and 73.3% in 2021), with a small increase in the participation of older adults in 2021. In 2012, 59.4% of participants lived with a partner; in 2021, 54.5% did not. In 2012, most of the sample belonged to classes A/B (46.4%), while in 2021 more than half belonged to class C (54.0%) (Table 1).

**Table 1.** Description of the sample with prevalence weighted according to sociodemographic and health characteristics. Pelotas, 2012 (n=2,925) and 2021 (n=5,717)

	Description of the sample	
Variables	2012 n (%)	2021 n (%)
Sex		
Male	1,202 (41.1)	1,898 (45.8)
Female	1,723 (58.9)	3,819 (54.2)
Age group (years)a		
20-29	612 (20.9)	724 (19.3)
30-39	540 (18.4)	865 (20.4)
40-49	594 (20.3)	923 (17.1)
50-59	514 (17.6)	1,097 (16.6)
60 or over	665 (22.8)	1,973 (26.7)
Race/skin colorb		
White	2,343 (80.1)	4,461 (77.5)
Black	354 (12.1)	856 (15.4)
Brown	192 (6.6)	348 (6.7)
Indigenous	19 (0.6)	6 (0.1)
Asian	16 (0.6)	13 (0.3)
Schooling (years)c		
Up to 4	524 (18.0)	8.25 (12.3)
5-8	817 (27.9)	1,578 (26.6)
9-11	819 (28.0)	1,879 (36.1)
12 or more	762 (26.1)	1,264 (25.1)
Economic classification (ABEP)d		
A/B (wealthiest)	1,348 (46.4)	1,337 (25.3)
C	1,261 (43.4)	3,034 (54.0)
D/E (poorest)	294 (10.2)	1,303 (20.8)
Marital statuse		
Does not have a partner	1,187 (40.6)	3,117 (54.5)
Has a partner	1,734 (59.4)	2,596 (45.5)
self-reported healthf		
Excellent/great/very good	903 (30.9)	683 (12.6)
Good	1,229 (42.0)	3,180 (57.5)
Fair	658 (22.5)	1,519 (25.1)
Poor/very poor	135 (4.6)	333 (4.9)

^a^ 135 values missing in 2021.

^b^ 1 value missing in 2012 and 33 values missing in 2021.

^c^ 2 values missing in 2012 and 171 values missing in 2021.

^d^ 22 values missing in 2012 and 43 values missing in 2021.

^e^ 4 values missing in 2012 and 2021; and

^f^ 2 values missing in 2021.

**Figure 1.**
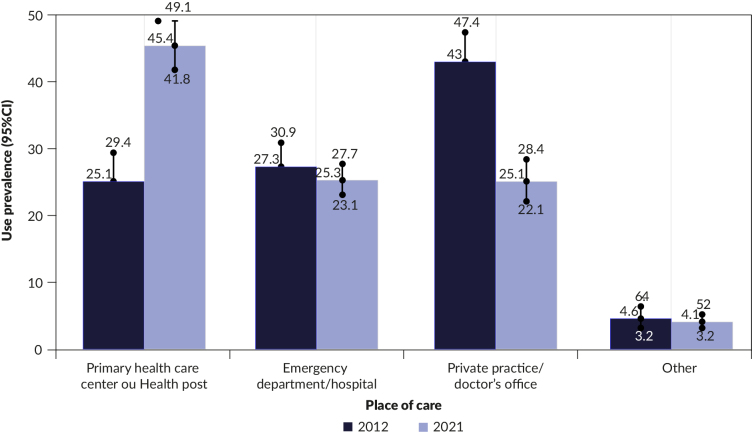
Weighted prevalence rates and confidence intervals (95%CI) of care provision services among people who used health services. Pelotas, 2012 (n=2,925) and 2021 (n=5,717)

Prevalence of health service use in the 30 days prior to the surveys was 29.3% (95%CI 27.6; 31.0) in 2012 and 34.4% (95%CI 32.5; 36.3) in 2021, which represented an increase of 5.1 percentage points. A significant increase of 20.4 percentage points was observed in the use of Primary health care center between 2012 (25.1%; 95%CI 21.3; 29.4) and 2021 (45.4%; 95%CI 41.8; 49.1). Use of private medical practices decreased significantly by 17.9 percentage points (43.0; 95%CI 38.7; 47.4 in 2012 versus 25.1; 95%CI 22.1; 28.4 in 2021) (Figure 1).

In both years, women (32.3; 95%CI 29.8; 34.7 in 2012 and 38.9; 95%CI 36.6; 41.1 in 2021) and individuals with poor/very poor self-rated health (61.1%; 95%CI 51.5; 70.7 in 2012 and 56.0%; 95%CI 50.1; 61.9 in 2021) used health services significantly more, even after adjusting for the variables. It was also found that, the greater the participants’ age, the higher the prevalence of use in both years. However, this association did not remain significant after adjusting for the other variables (Table 2).

There was positive association between education level and use of health services, with higher prevalence found among those with 12 or more years of education (35.2; 95%CI 30.9; 39.5 in 2012 and 37.7; 95%CI 33.6; 41.8 in 2021), although it was only statistically significant in 2012. Individuals in class D/E showed higher prevalence of use in 2021 (37.9%; 95%CI 34.0; 41.7; p-value 0.010) (Table 2).

**Table 2.** Unadjusted and adjusted weighted prevalence rates and 95% confidence intervals (95%CI) of health service use according to sociodemographic and health characteristics. Pelotas, 2012 (n=2,925) and 2021 (5,717)

Variables	Health service use		
	2012		2021		
	Unadjusted % (95%CI)	Adjusted % (95%CI)	Unadjusted % (95%CI)	Adjusted % (95%CI)
Sex	p-value<0.001	p-value<0.001	p-value<0.001	p-value<0.001
Male	22.7 (20.4; 25.2)	23.9 (21.4; 26.6)	28.2 (25.9; 30.6)	29.1 (26.4; 31.8)
Female	33.8 (31.4; 36.4)	32.3 (29.8; 34.7)	39.6 (37.2; 42.2)	38.9 (36.6; 41.1)
Age group (years)	p-value 0.021^a^	p-value 0.721^a^	p-value 0.005^a^	p-value 0.063^a^
20-29	26.5 (23.0; 30.3)	29.1 (25.0; 33.2)	33.3 (29.3; 37.6)	34.1 (29.6; 38.6)
30-39	25.7 (22.5; 29.2)	27.4 (23.8; 30.9)	31.9 (28.4; 35.6)	32.2 (28.6; 35.8)
40-49	30.0 (26.3; 34.0)	30.3 (26.6; 34.0)	32.1(28.7; 35.6)	32.3 (28.8; 35.9)
50-59	29.8 (25.7; 34.2)	29.1 (25.0; 33.2)	36.0 (32.6; 39.4)	35.7 (32.4; 38.9)
60 or over	33.7 (30.0; 37.5)	29.3 (25.5; 33.0)	38.5 (35.8; 41.2)	37.4 (34.8; 40.0)
Race/skin color	p-value 0.813	p-value 0.684	p-value 0.490	p-value 0.534
White	29.0 (27.1; 31.0)	28.7 (26.8; 30.6)	33.8 (31.8; 35.9)	34.1 (32.0; 36.3)
Black	30.2 (25.5; 35.4)	30.7 (25.6; 35.9)	35.3 (31.3; 39.4)	34.8 (30.9; 38.6)
Brown	29.2 (23.7; 35.3)	29.5 (23.2; 35.9)	38.5 (33.4; 43.9)	38.9 (33.6; 44.2)
Indigenous	42.1 (23.6; 63.2)	39.3 (20.8; 57.8)	28.8 (8.2; 64.5)	18.4 (7.5; 54.3)
Asian	37.5 (17.8; 62.5)	31.2 (11.5; 50.9)	40.6 (14.0; 74.2)	38.3 (5.5; 71.1)
Schooling (years)	p-value 0.023^a^	p-value<0.001^a^	p-value 0.143^a^	p-value 0.059^a^
Up to 4	33.2 (29.6; 37.0)	25.8 (22.5; 29.2)	36.3 (31.7; 41.1)	30.7 (26.1; 35.2)
5-8	26.1 (23.4; 29.0)	25.0 (22.2; 27.8)	36.5 (33.2; 40.0)	34.3 (31.1; 37.4)
9-11	29.9 (26.8; 33.3)	31.3 (27.8; 34.8)	32.7 (30.3; 35.1)	34.4 (31.9; 36.8)
12 or more	29.1 (26.0; 32.5)	35.2 (30.9; 39.5)	33.0 (29.9; 36.2)	37.7 (33.6; 41.8)
Economic classification (ABEP)	p-value 0.559^a^	p-value 0.955^a^	p-value<0.001^a^	p-value 0.010^a^
A/B (wealthiest)	28.0 (25.6; 30.7)	28.8 (26.1; 31.4)	30.3 (27.7; 32.9)	31.6 (28.3; 34.9)
C	29.9 (27.3; 32.7)	29.7 (26.9; 32.6)	34.4 (32.3; 36.5)	34.6 (32.5; 36.8)
D/E (poorest)	30.3 (25.4; 35.6)	27.7 (22.6; 32.9)	40.0 (36.2; 44.0)	37.9 (34.0; 41.7)
Marital status	p-value 0.198	p-value 0.925	p-value 0.210	p-value 0.760
Does not have a partner	30.7 (27.9; 33.8)	29.0 (26.3; 31.7)	33.6 (31.4; 35.9)	34.8 (32.3; 37.3)
Has a partner	28.3 (26.1; 30.5)	29.1 (27.0; 31.3)	35.3 (32.9; 37.8)	34.3 (32.3; 37.3)
Self-rated health	p-value<0.001	p-value<0.001	p-value<0.001	p-value<0.001
Excellent/great/very good	22.0 (19.4; 24.9)	21.4 (18.6; 24.2)	24.0 (20.4; 28.0)	24.1 (20.1; 28.2)
Good	25.4 (23.1; 27.8)	25.2 (23.0; 27.4)	31.1 (28.9; 33.4)	31.7 (29.4; 34.1)
Fair	40.2 (36.6; 44.0)	41.3 (37.0; 45.6)	42.4 (39.2; 45.6)	41.4 (38.3; 44.6)
Poor/very poor	59.2 (50.7; 67.3)	61.1 (51.5; 70.7)	59.3 (53.4; 65.0)	56.0 (50.1; 61.9)

^a^ Trend test.

Brazilian Association for Population Studies

**Figure 2.**
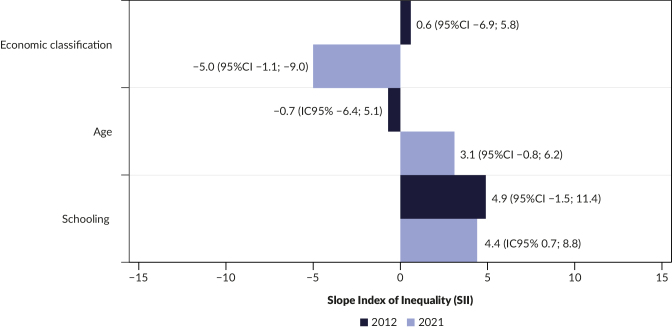
Absolute inequalities in health service use according to economic classification, schooling and age variables. Pelotas, 2012 (n=2,925) and 2021 (n=5,717)

In 2012, 54.3% of care involved payment via health insurance or private services. Among those who used private services the most, there were predominantly women (67.1%), those aged 20-49 years (57.2%), White individuals (84.3%), those with a higher level of education (40.3%), those who had a partner (59.3%), were wealthier (67.7%), and had good self-rated health (41.3%). In 2021, 69.8% of the cost of care was borne by the SUS. Public service users included women (63.2%), people aged 20-49 years (56.7%), White individuals (72.6%), those with 9 to 11 years of education (35.4%), those who had a partner (52.4%), those belonging to economic class C (54.1%), and those with good self-rated health (50.7%) (Table 3).

Regarding absolute inequalities, there was an increase in differences in terms of income (2012: 0.6; 95%CI -6.9; 5.8 and 2021: -5.0; 95%CI -1.1; -9.0) and a slight reduction according to education level from 2012 (4.9; 95%CI 1.5; 11.4) to 2021 (4.4; 95%CI 0.7; 8.8), while the difference related to age increased (2012: -0.7; 95%CI -6.4; 5.1 and 2021: 3.1; 95%CI -0.8; 6.2), but was not significant in either year. Overall, in 2021, service use increased among the poorest and most educated individuals compared to 2012 (Figure 2).

Sensitivity analyses performed for 2012 showed similar results, considering both all household residents and just one selected household resident, with an increase in the prevalence of use from 29.3% (95%CI 27.6; 31.0) to 30.7% (95%CI 28.2; 33.3). Compared to 2021, analyses performed without weighting the sample design indicated an increase in the prevalence of use from 34.4% (95%CI 32.5; 36.3) to 36.5% (95%CI 34.5; 38.4), maintaining a similar pattern of inequalities.

**Table 3:** Description of sociodemographic and health characteristics according to service cost payment source among people who used health services. Pelotas, 2012 (n=856) and 2021 (n=2,085)

	Service cost payment source		
	2012		2012	
Variables	Public n (%)	Private n (%)	Public n (%)	Private n (%)
Sex				
Male	120 (30.7)	153 (32.9)	379 (36.8)	174 (39.3)
Female	271 (69.3)	312 (67.1)	1,065 (63.2)	467 (60.7)
Age group (years)				
20-29	63 (16.1)	99 (21.3)	193 (20.0)	53 (15.3)
30-39	65 (16.6)	74 (15.9)	227 (19.9)	74 (16.2)
40-49	85 (21.7)	93 (20.0)	228 (16.8)	87 (13.6)
50-59	75 (19.2)	78 (16.8)	298 (18.0)	116 (15.5)
60 or over	103 (26.3)	121 (26.0)	464 (25.4)	304 (39.4)
Race/skin color				
White	287 (73.4)	392 (84.3)	1,052 (72.6)	553 (84.7)
Black	61 (15.6)	46 (9.9)	262 (18.6)	54 (9.3)
Brown	36 (9.2)	20 (4.3)	112 (8.4)	31 (5.5)
Indigenous	5 (1.3)	3 (0.9)	2 (0.1)	0 (0.0)
Asian	2 (0.5)	4 (0.6)	4 (0.3)	2 (0.4)
Schooling (years)^a^				
Up to 4	120 (20.4)	54 (11.6)	65 (15.0)	247 (8.4)
5-8	142 (24.9)	71 (15.3)	112 (33.5)	496 (16.7)
9-11	93 (28.7)	152 (32.8)	203 (35.4)	454 (32.3)
12 or over	35 (26.0)	187 (40.3)	249 (16.1)	184 (42.6)
Economic classification (ABEP)^b^				
A/B (wealthiest)	81 (21.0)	297 (67.7)	183 (14.5)	249 (39.9)
C	237 (61.6)	140 (30.5)	764 (54.1)	331 (52.8)
D/E (poorest)	67 (17.4)	22 (4.8)	490 (31.4)	57 (7.3)
Marital status^c^				
Does not have a partner	176 (45.0)	189 (40.7)	688 (47.6)	282 (44.9)
Has a partner	215 (55.0)	275 (59.3)	754 (52.4)	359 (55.1)
Self-reported health				
Excellent/great/very good	53 (13.6)	146 (31.4)	108 (7.8)	67 (11.0)
Good	120 (30.7)	192 (41.3)	693 (50.7)	340 (54.7)
Fair	158 (40.4)	107 (23.0)	489 (32.2)	186 (28.1)
Poor/very poor	60 (15.4)	20 (4.3)	154 (9.3)	48 (6.2)

^a^ 2 values missing in 2012 and 171 values missing in 2021;

^b^ 22 values missing in 2012 and 43 values missing in 2021; and

^c^ 4 values missing in 2012 and 2 values missing in 2021. Brazilian Association for Population Studies.

## Discussion

From 2012 to 2021, prevalence of health service use increased from 29.3% to 34.4%. This increase was markedly accentuated by the use of Primary health care center in 2021, rather than doctors’ consulting rooms in 2012. Consequently, there was also greater use of services funded by the SUS in 2021. The sociodemographic profile of public and private service users in the years assessed differed, although there was a higher proportion of adults between 20 and 49 years old in both situations. In terms of absolute inequality, income-related inequality increased, and there was a slight reduction in differences according to education level.

Analyses of population surveys conducted in São Paulo in 2003, 2008, and 2015 revealed increases in service demand, use and delivery, increased use of the SUS, and changes in patterns of educational inequalities over time ^19^ ^,^ ^20^. Although conducted in earlier periods and in different local contexts, these results were consistent with those of this study, which indicated increased use of public services by more socially vulnerable groups, reinforcing the importance of the public health system in promoting equity.

This upward trend in SUS use, observed over time, was abruptly interrupted with the advent of the COVID-19 pandemic, which significantly impacted health service supply and demand across the country. Previous national analyses identified a reduction in demand or supply of services during the pandemic period ^4^ ^-^ ^6^; however, the data presented here show an increase in service use between 2012 and 2021.

One possible explanation for this result lies in the data collection period for the 2021 survey, which occurred after the most critical phase of the pandemic. Many studies focused specifically on 2020, when health services were more restricted and the population avoided seeking care for fear of infection. The data analyzed here reflect a later period, marked by the beginning of the vaccination campaign and greater reorganization of services. The fact that COVID-19 vaccination was implemented primarily within the SUS may also have stimulated demand for public services ^21^. Furthermore, the results may indicate a more structured local response by the SUS in Pelotas, given the accumulated demands of the population and the worsening social inequalities in the context of the pandemic.

The increased use of public services during this period may also reflect socioeconomic inequalities, stemming from the hostile sociocultural and political context that affects Latin American countries. Historical processes of colonization and enslavement, which are reflected in the division of resources to this day, were marked and exacerbated during the COVID-19 pandemic ^22^.

Two main points can explain the results of this study: the first concerns how the pandemic crisis amplified the economic crisis that began in Brazil in 2016 and fueled the intensification of poverty in the country ^22^ ^,^ ^23^. For example, poverty rates rose sharply in 2020 ^23^, and this can also be seen from this study, which shows that, overall, there was a reduction in the socioeconomic status of the population from 2012 to 2021. In the period compared, there was a reduction of just over 20 percentage points in classes A/B and an increase of about 10 percentage points in classes C and D/E.

The second point concerns how COVID-19-related measures, such as social distancing (lockdowns), impacted workers, with approximately 10 million people leaving the workforce between the third quarter of 2019 and the third quarter of 2020 ^23^. The two points appear to be intrinsically related. The negative economic effects of the pandemic particularly affected women, young people and the less educated, who were also more likely to lose their jobs during the pandemic ^23^ ^,^ ^24^.

In this study, we observed how the younger population, which in 2012 was usually served by private or paid services, began to be served by services provided by the SUS in 2021. Data on health insurance coverage in Pelotas, in December 2012 and 2021, revealed that coverage decreased from 16.3% to 15.8% ^25^. This result suggests reflection on how unemployment may have affected this population, including possible loss of health insurance due to inability to pay, resulting in the need to use exclusively public services ^23^ ^,^ ^24^. In the case of education, the explanation tends to be the same, although inequalities were not statistically significant.

This study compared data from two distinct periods, nine years apart, within different political, economic and health contexts. These changes over time could influence the pattern of healthcare service use. Furthermore, there were significant methodological differences between the studies, which also limited direct comparability between the periods. In 2012, all residents aged 10 or older in the household were interviewed, while in 2021, only one resident was selected. Although this difference alone does not explain the sample profile, a higher proportion of women and older adults than expected was found in 2021.

In order to mitigate this potential selection bias, a post-stratification strategy based on population projections by sex and age was applied. Sensitivity tests were performed to assess the impact of these methodological and weighting differences, and the results remained consistent, reinforcing the robustness of the main findings. Sampling weights were also not harmonized between the data collections, which may limit the direct comparability of absolute inequality measures between periods.

Another limitation was the exclusion of certain variables, such as presence of multimorbidity, from the adjusted analyses, since it is considered a potential mediator in the relationship between sociodemographic factors and health service use. The use of the slope index of inequality allowed for a comprehensive analysis of health inequalities, considering the entire distribution of socioeconomic variables, not just the extreme categories. However, as it is a summarized measure, it may mask significant variations between intermediate categories.

The findings of this study revealed an increase in the use of health services, especially among the most vulnerable populations. Although inequalities still persist, the increased use of public services in 2021 reflected not only an emergency response to economic hardship but also the importance of the SUS in times of crisis. This leads to reflection on the need to strengthen the health system, ensuring its accessibility to all, especially those groups most affected by socioeconomic inequalities.

## Data Availability

The data used in this research are not available in public repositories, but can be accessed upon formal request. The Master’s Consortium database (2012) can be requested from the Board of the Federal University of Pelotas Epidemiology Postgraduate Program. Data from the EAI Pelotas study (2021) can be requested using a form available at: https://wp.ufpel.edu.br/eaipelotas/banco-de-dados/. The questionnaires are available at the respective websites: https://guaiaca.ufpel.edu.br/handle/123456789/1952; and https://wp.ufpel.edu.br/eaipelotas/questionario/.

## References

[1] Travassos C, Castro MSM (2012). Políticas e sistema de saúde no Brasil.

[2] Castro MC, Massuda A, Almeida G, Menezes-Filho NA, Andrade MV, Souza Noronha KVM (2019). Brazil’s unified health system: the first 30 years and prospects for the future.. Lancet.

[3] Oliveira MM, Fuller TL, Gabaglia CR, Cambou MC, Brasil P, Vasconcelos ZFM (2022). Repercussions of the COVID-19 pandemic on preventive health services in Brazil. Prev Med..

[4] Mehrotra A, Chernew M, Linetsky D, Hatch H, Cutler D, Schneider E (2020). The impact of the COVID-19 pandemic on outpatient care: Visits return to prepandemic levels, but not for all providers and patients. Commonwealth Fund.

[5] Chisini LA, Castilhos ED, Costa FS, D’Avila OP (2021). Impact of the COVID-19 pandemic on prenatal, diabetes and medical appointments in the Brazilian National Health System. Rev Bras Epidemiol.

[6] Horta BL, Silveira MF, Barros AJD, Hartwig FP, Dias MS, Menezes AMB (2022). COVID-19 and outpatient care: a nationwide household survey. Cad Saude Publica.

[7] Stahnke DN, Medeiros BM, Martins RB, Costa JSD (2024). Tendência de internações por condições sensíveis à atenção primária em Pelotas, Brasil, de 2000 a 2021. Cien Saude Colet.

[8] Könsgen BI, Nunes BP, Facchini LA, Tomasi E (2021). Utilização de serviços de saúde e fatores associados, entre estudantes da Universidade Federal de Pelotas: estudo transversal, 2018. Epidemiol Serv Saude.

[9] Nunes BP, Flores TR, Duro SMS, Saes MO, Tomasi E, Santiago AD (2015). Utilização dos serviços de saúde por adolescentes: estudo transversal de base populacional, Pelotas-RS, 2012. Epidemiol Serv Saude.

[10] Nunes BP, Thumé E, Tomasi E, Duro SMS, Facchini LA (2014). Socioeconomic inequalities in the access to and quality of health care services. Rev Saude Publica.

[11] Bertoldi AD, Silveira MPT, Machado AKF, Xavier MO, Martins RC (2021). Fontes de acesso e utilização de medicamentos na zona rural de Pelotas, Rio Grande do Sul, em 2016: estudo transversal de base populacional. Epidemiol Serv Saude.

[12] Neves RG, Duro SMS, Tomasi E. (2016). Vacinação contra influenza em idosos de Pelotas-RS, 2014: um estudo transversal de base populacional. Epidemiol Serv Saude.

[13] Tasca R, Martins MBC, Malik AM, Schiesari LMC, Bigoni A, Costa CF (2022). Gerenciando o SUS no nível municipal ante a Covid-19: uma análise preliminar. Saúde em Debate.

[14] Barros AJD, Menezes AMB, Santos IS, Assunção MCF, Gigante D, Fassa AG (2008). O Mestrado do Programa de Pós-graduação em Epidemiologia da UFPel baseado em consórcio de pesquisa: uma experiência inovadora. Rev Bras Epidemiol.

[15] Nunes BP (2013). Acesso aos serviços de saúde em adolescentes e adultos na cidade de Pelotas-RS.

[16] Delpino FM, Figueiredo LM, Costa ÂK, Carreno I, Silva LN, Flores AD (2023). Emergency department use and Artificial Intelligence in Pelotas: design and baseline results. Rev Bras Epidemiol.

[17] Associação Brasileira de Empresas de Pesquisa (2009). Critério de classificação econômica Brasil.

[18] Barros AJ, Victora CG (2013). Measuring coverage in MNCH: determining and interpreting inequalities in coverage of maternal, newborn, and child health interventions. PLoS Med.

[19] Santos EFS, Louvison MCP, Oliveira ECT, Monteiro CN, Barros MBA, Goldbaum M (2023). Analysis of education level in access and use of health care services, ISA-Capital, São Paulo, Brazil, 2003 and 2015. Cad Saude Publica.

[20] Monteiro CN, Beenackers MA, Goldbaum M, Barros MBA, Gianini RJ, Cesar CLG (2017). Use, access, and equity in health care services in São Paulo, Brazil. Cad Saude Publica.

[21] Maciel E, Fernandez M, Calife K, Garrett D, Domingues C, Kerr L (2022). A campanha de vacinação contra o SARS-CoV-2 no Brasil e a invisibilidade das evidências científicas. Cien Saude Colet.

[22] Economic Commission for Latin America and the Caribbean (ECLAC) (2023). Social Panorama of Latin America and the Caribbean.

[23] World Bank Group. Brazil Poverty and Equity Assessment Looking ahead of two crises (2022). https://openknowledge.worldbank.org/server/api/core/bitstreams/19298bfa-067d-504c-8e34-00b20e3139d2/content.

[24] Costa SS (2020). Pandemia e desemprego no Brasil. Rev Adm Pública.

[25] TabNet Linux 2.6a: Beneficiários por Município.

