# Supplementary material for: Adult use of health services: A cross-sectional population-based study, Pelotas, Brazil, 2012 and 2021
Source: Epidemiol Serv Saude. 2026 Apr 20;35:e20250460. doi: 10.1590/S2237-96222026v35e20250460.en (PMC13102448; doi:10.1590/S2237-96222026v35e20250460.en)
Supplement: Translation

# Translation: Utilização dos serviços de saúde entre adultos: estudo transversal de base populacional, Pelotas, 2012 e 2021

**DOI:** 10.1590/S2237-96222026v35e20250460.pt

**Authors:** Larissa Adna Neves Silva, Felipe Mendes Delpino, Lilian Munhoz Figueiredo, Elaine Thumé, Elaine Tomasi, Luiz Augusto Facchini, Bruno Pereira Nunes

**Affiliations:** 1 Universidade Federal de Pelotas, Pós-Graduação em epidemiologia, Pelotas, RS, Brasil; 2 Universidade Federal de Pelotas, Programa de Pós-Graduação em Odontologia, Pelotas, RS, Brasil; 3 Universidade Federal de Pelotas, Pós-Graduação em Enfermagem, Pelotas, RS, Brasil

**Keywords:** Serviços de Saúde, Desigualdades em Saúde, COVID-19, Adultos, Estudos Transversais

## Abstract

**Objetivos:**

Comparar a prevalência de utilização dos serviços de saúde entre adultos da zona urbana de Pelotas, em 2012 e 2021, e avaliar as desigualdades socioeconômicas nesses anos.

**Métodos:**

Trata-se de estudo transversal de base populacional com adultos residentes na zona urbana de Pelotas em 2012 e 2021. Foram realizadas análises brutas e ajustadas, por meio da regressão de Poisson, da utilização de serviços de saúde nos 30 dias anteriores às pesquisas de acordo com sexo, idade, escolaridade, raça/cor da pele, classe econômica, situação conjugal e percepção de saúde. O índice absoluto de desigualdade foi utilizado para mensurar desigualdades.

**Resultados:**

A amostra foi composta por 2.925 participantes em 2012 e 5.717 em 2021. A prevalência de utilização de serviços de saúde aumentou de 29,3% (intervalo de confiança de 95% - IC95% 27,6; 31,1) para 34,4% (IC95% 33,2; 35,6). Entre os usuários, a utilização de unidades do Sistema Único de Saúde cresceu de 45,7% (IC95% 42,7; 48,7) para 69,8% (IC95% 67,8; 71,7), com redução proporcional no uso de serviços particulares. Em 2021, observou-se maior dependência dos serviços públicos entre mulheres (63,2%), mais pobres (31,4%) e indivíduos mais jovens (56,7%). Houve aumento nas desigualdades por classe econômica.

**Conclusão:**

Houve aumento na utilização dos serviços de saúde e maior dependência dos serviços públicos em 2021, especialmente entre grupos socialmente vulneráveis. As desigualdades socioeconômicas se acentuaram, o que refletiu mudanças no padrão de acesso em um contexto de crise econômica e sanitária.

**Aspectos éticos**

Esta pesquisa respeitou os princípios éticos, obtendo os seguintes dados de aprovação:

Comitê de ética em pesquisa: Faculdade de Medicina da Universidade Federal de Pelotas

Número do parecer: Protocolo nº 77/11

Data de aprovação: 1/12/2011

Comitê de ética em pesquisa:Faculdade de Medicina da Universidade Federal de Pelotas

Número do parecer: 39096720.0.0000.5317

Data de aprovação: 10/1/2021

Registro do consentimento livre e esclarecido: Obtido de todos os participantes.

Uso de inteligência artificial generativa

Os pesquisadores utilizaram o ChatGPT (https://chatgpt.com/), com a finalidade de revisar a gramática da seção *Discussão* e garantir maior clareza.

## Introdução 

Os sistemas de saúde universais desempenham papel crucial na redução das desigualdades sociais e na garantia do acesso aos serviços de saúde. O acesso se refere à relação entre a procura e a entrada do indivíduo no sistema de saúde e depende da percepção de necessidade, das características socioeconômicas e da disponibilidade dos serviços ^(^ ^1^ ^)^.

Apesar dos desafios desde sua criação na década de 1990, o Sistema Único de Saúde (SUS) melhorou a equidade em saúde em todo o Brasil, visto que ampliou o acesso aos serviços de saúde a partir da expansão da Atenção Primária à Saúde por meio da Estratégia de Saúde da Família. No entanto, desde 2016 com a implementação de políticas de austeridade fiscal ^(^ ^2^ ^)^ e com o início e decorrer da pandemia de coronavírus, essas conquistas foram impactadas negativamente, com ameaças constantes sobre o fornecimento de serviços e a garantia de acesso à saúde ^(^ ^2^ ^,^ ^3^ ^)^. 

Entre 2020 e 2021, em países de alta renda, como Estados Unidos, Canadá, Reino Unido e Austrália, houve quedas expressivas nas visitas ambulatoriais para consultas de rotina, triagem de doenças e cuidados preventivos, principalmente no início da pandemia da covid-19 ^(^ ^4^ ^)^. No Brasil, durante 2020, observaram-se declínios significativos na disponibilidade de consultas médicas e odontológicas e na realização de procedimentos envolvendo cuidados pré-natais e a pessoas com diabetes ^(^ ^3^ ^,^ ^5^ ^)^. Nesse período, cerca de um em cada quatro indivíduos não procurou atendimento devido a um problema de saúde ou faltou a um exame ^(^ ^6^ ^)^.

Em 2020, Pelotas, com cerca de 350 mil habitantes, possuía 62% da população coberta por equipes de saúde da família - aumento de 28 pontos percentuais comparado a 2012 quando a cobertura era apenas 34% ^(^ ^7^ ^)^. Entre 2012 e 2021, o município passou por mudanças relevantes na estrutura da atenção primária, o que incluiu reorganizações de equipes e redirecionamento dos atendimentos durante a pandemia, com impacto direto sobre a oferta e a forma de acesso da população aos serviços ^(^ ^7^ ^-^ ^10^ ^)^. A ampliação da cobertura não eliminou as desigualdades no acesso aos serviços de saúde, amplamente descritas ^(^ ^10^ ^-^ ^12^ ^)^ e intensificadas durante a pandemia ^(^ ^13^ ^)^. 

Como atrasos no atendimento e falta de acesso podem ter consequências de curto e longo prazo na saúde da população, é importante avaliar as mudanças que ocorreram no acesso aos serviços. Os objetivos deste artigo foram comparar a prevalência de utilização dos serviços de saúde entre adultos da zona urbana de Pelotas, Rio Grande do Sul, em 2012 e 2021, e avaliar as desigualdades socioeconômicas nesses anos. 

## Métodos

 Desenho do estudo 

Trata-se de estudo transversal que utilizou dados de duas pesquisas transversais de base populacional realizadas em 2012 e 2021 na zona urbana de Pelotas, Rio Grande do Sul. 

Contexto

 A primeira pesquisa foi conduzida durante um consórcio de mestrado do Programa de Pós-Graduação em Epidemiologia da Universidade Federal de Pelotas em 2012 ^(^ ^14^ ^)^. No âmbito do consórcio, cada mestrando realizou o cálculo do tamanho de amostra com base no desfecho específico a ser investigado, incorporando acréscimos de 10% para possíveis perdas e recusas e de 15% para controle de fatores de confusão e para o ajuste do efeito de delineamento. A maior amostra estimada foi de 3.120 indivíduos adultos e 800 adolescentes. Para alcançar esse quantitativo, foi estimada a necessidade de incluir 1.560 domicílios no município. Para considerar o efeito do desenho amostral, foram sorteados 130 setores censitários, prevendo-se a visita a 12 domicílios por setor ^(^ ^15^ ^)^. 

Em 2012, o município contava com 495 setores censitários, conforme o Censo Demográfico de 2010 do Instituto Brasileiro de Geografia e Estatística. Devido à indisponibilidade de informações sobre o nível socioeconômico dos setores, estes foram ordenados segundo sua numeração original. A partir desse ordenamento, realizou-se uma amostragem sistemática. Cada setor dispunha de informações quanto ao número total de domicílios, identificados por um número inicial e um número final, o que totalizou 107.152 domicílios no município ^(^ ^15^ ^)^. 

Esse total foi dividido pelos 130 setores a serem selecionados, definindo-se o intervalo sistemático de 824 domicílios. A seleção dos setores foi iniciada a partir do número aleatório 634, sorteado no programa Stata, e realizada de forma sistemática, respeitando a probabilidade proporcional ao número de domicílios em cada setor. Em cada domicílio selecionado, todos os indivíduos de 10 anos ou mais de idade foram convidados a participar do estudo. 

Foram realizadas 3.671 entrevistas, com 13,4% de perdas e recusas. Em casos de recusa, o domicílio não foi substituído. Consideraram-se como perdas e recusas as entrevistas não realizadas após três tentativas em dias e horários diferentes, sendo uma realizada por um supervisor do estudo. Mais detalhes do processo amostral podem ser obtidos em outras publicações ^(^ ^15^ ^)^. 

A segunda pesquisa aconteceu em 2021, com o projeto de coorte intitulado Emergency department use and Artificial Intelligence in Pelotas (EAI PELOTAS). Neste trabalho, foram utilizados os dados de linha de base coletados entre setembro e dezembro de 2021. O processo de amostragem também foi realizado em múltiplos estágios, o primeiro consistiu na seleção aleatória de 100 setores censitários com base em estimativas atualizadas pelo Instituto Brasileiro de Geografia e Estatística em 2019. Depois, foi definido um número proporcional de domicílios para cada setor censitário. Por fim, foi entrevistado um morador de 18 anos ou mais em cada domicílio; caso houvesse mais de um morador elegível, a escolha era feita por um aplicativo gerador de números aleatórios ^(^ ^16^ ^)^. Mais detalhes sobre a pesquisa podem ser encontrados em outra publicação ^(^ ^16^ ^)^ e na página do estudo na internet: https://wp.ufpel.edu.br/eaipelotas/.

Participantes

No inquérito de 2012, todos os moradores elegíveis (com 20 anos ou mais) de cada domicílio selecionado foram incluídos na análise, enquanto em 2021 apenas um morador por domicílio foi entrevistado e este foi incluído na análise.

Variáveis e mensuração

O desfecho foi a utilização de serviços de saúde definida como a proporção de indivíduos que procuraram atendimento e foram atendidos. Em ambos os estudos, a pergunta utilizada foi “Desde (dia do mês passado), o(a) Sr. (a) foi atendido em algum serviço de saúde”? Dessa forma, o período de referência correspondeu aos 30 dias anteriores à entrevista. 

As variáveis independentes selecionadas foram: sexo (masculino, feminino), idade em anos completos (20-29, 30-39, 40-49, 50-59, 60+), raça/cor da pele (branca, preta, parda, amarela, indígena), escolaridade em anos completos (até 4, 5-8, 9-11, 12+), situação conjugal (sem companheiro, com companheiro), classificação econômica operacionalizada pela Associação Brasileira de Empresas de Pesquisa - ABEP ^(^ ^17^ ^)^ (A - mais rico e B, C, D e E - mais pobre e categorizada, A/B, C, D/E) e percepção de saúde (excelente/ótima/muito boa, boa, regular, ruim/péssima). As variáveis de classificação econômica, idade e escolaridade foram utilizadas para estimativa das desigualdades, conforme descrito na subseção *Métodos estatísticos*.

A percepção de saúde foi avaliada por meio da pergunta “Como o(a) Sr.(a) considera sua saúde?” em ambos os inquéritos. A classificação econômica de acordo com a ABEP, ano de 2008, foi realizada conforme a posse de determinados bens e serviços (como televisão em cores, rádio, banheiro, automóvel, máquina de lavar, DVD, geladeira e freezer), além da presença de empregada doméstica mensalista e do grau de escolaridade do chefe do domicílio. Cada item possuiu uma pontuação específica, que foi somada para gerar um escore final. A categorização foi realizada de acordo com os pontos de corte indicados no documento da associação ^(^ ^17^ ^)^.

Para caracterização do último atendimento recebido no mês anterior à entrevista, foram utilizadas as variáveis: natureza jurídica do serviço de saúde (SUS, particular/por algum convênio) e local de atendimento (posto de saúde, pronto-socorro/pronto-atendimento/hospital, consultório médico, outros). A categoria outros correspondeu a serviços relacionados ao centro de especialidades, centro de atenção psicossocial e serviços de saúde de outras cidades. 

Métodos estatísticos

Foram realizadas análises descritivas com cálculos de prevalência e respectivos intervalos de confiança de 95% (IC95%). As diferenças nas prevalências foram avaliadas por meio do teste de heterogeneidade e, quando aplicável, do teste de tendência. Após a descrição das análises bivariadas, calcularam-se as prevalências ajustadas e seus respectivos IC95% por meio de modelo de regressão de Poisson, com ajuste mútuo entre as variáveis. Todas as variáveis foram adicionadas ao mesmo tempo. As estimativas ajustadas foram obtidas por meio de pós-estimação*,* que permitiu calcular as probabilidades preditas da variável dependente para cada categoria das variáveis independentes, mantendo as demais constantes. 

Valores de p>0,05 foram considerados estatisticamente significativos. Todas as análises foram realizadas no software Stata, versão 18.0, e levaram em consideração o desenho amostral complexo por meio da definição prévia do plano amostral.

Para avaliar as desigualdades, foi utilizado o índice absoluto de desigualdade, calculado por meio de regressão logística para variáveis categóricas ordinais, como idade, escolaridade e classificação econômica. O índice expressou a diferença absoluta ajustada na probabilidade de utilização dos serviços de saúde entre os valores mais baixos e mais altos da variável analisada - como os indivíduos mais jovens em comparação aos mais velhos, ou os menos escolarizados em comparação aos mais escolarizados - considerando toda a distribuição dos grupos intermediários. Valor igual a zero indicou ausência de desigualdade. Valores positivos indicaram maior utilização dos serviços nos grupos mais velhos, com maior escolaridade ou em melhores condições socioeconômicas. Valores negativos indicaram maior utilização entre os mais jovens, com menor escolaridade ou em situação social mais desfavorável ^18^. 

Também foram realizadas duplas estratificações, ou seja, as análises foram feitas considerando simultaneamente duas variáveis: a natureza jurídica dos serviços de saúde e outras variáveis independentes, como escolaridade, idade e classificação econômica. Por exemplo, analisou-se se a utilização dos serviços públicos ou privados variava entre grupos com diferentes níveis de escolaridade ou faixas etárias ou ainda classes econômicas. 

Foram realizadas análises de sensibilidade com o objetivo de avaliar a robustez e a consistência dos achados principais. As análises incluíram: i) a reanálise dos dados de 2012 considerando apenas um morador por domicílio, a fim de padronizar a unidade de análise e possibilitar a comparabilidade com os dados de 2021, que foram coletados com base em um único indivíduo por domicílio; e ii) a avaliação dos dados de 2021 sem a aplicação da estratégia de pós-estratificação, com o intuito de examinar o impacto da ponderação na correção de potenciais vieses de seleção decorrentes do processo amostral. 

## Resultados

Em 2012 e 2021, foram avaliados 2.925 e 5.717 indivíduos adultos e idosos. Em ambos os anos, pouco mais da metade da população estudada era do sexo feminino (58,9% em 2012 e 54,2% em 2021), a maioria se identificava como raça/cor da pele branca (80,1% em 2012 e 77,5% em 2021), possuía entre 9 e 11 anos de escolaridade (28,0% em 2012 e 36,1% em 2021) e relatava boa percepção de saúde (42,0% em 2012 e 57,5% em 2021). A maior parte dos participantes estava na faixa etária 20-59 anos (77,2% em 2012 e 73,3% em 2021), com um pequeno aumento na participação de idosos em 2021. Em 2012, 59,4% dos participantes viviam com companheiro; em 2021, 54,5% não estavam nessa condição. Em 2012, a maior parte da amostra pertencia às classes A/B (46,4%), enquanto em 2021 mais da metade era da classe C (54,0%) (Tabela 1).

**Tabela 1:** Descrição da amostra com prevalências ponderadas de acordo com as características sociodemográficas e de saúde. Pelotas, 2012 (n=2.925) e 2021 (n=5.717)

Variáveis	Descrição da amostra	
	2012 n (%)	2021 n (%)
Sexo		
Masculino	1.202 (41,1)	1.898 (45,8)
Feminino	1.723 (58,9)	3.819 (54,2)
Faixa etária (anos)^a^		
20-29	612 (20,9)	724 (19,3)
30-39	540 (18,4)	865 (20,4)
40-49	594 (20,3)	923 (17,1)
50-59	514 (17,6)	1.097 (16,6)
60 ou mais	665 (22,8)	1.973 (26,7)
Raça/cor da pele^b^		
Branca	2.343 (80,1)	4.461 (77,5)
Preta	354 (12,1)	856 (15,4)
Parda	192 (6,6)	348 (6,7)
Indígena	19 (0,6)	6 (0,1)
Amarela	16 (0,6)	13 (0,3)
Escolaridade (anos)^c^		
Até 4	524 (18,0)	8.25 (12,3)
5-8	817 (27,9)	1.578 (26,6)
9-11	819 (28,0)	1.879 (36,1)
12 ou mais	762 (26,1)	1.264 (25,1)
Classificação econômica (ABEP)^d^		
A/B (mais ricos)	1.348 (46,4)	1.337 (25,3)
C	1.261 (43,4)	3.034 (54,0)
D/E (mais pobres)	294 (10,2)	1.303 (20,8)
Situação conjugal^e^		
Sem companheiro	1.187 (40,6)	3.117 (54,5)
Com companheiro	1.734 (59,4)	2.596 (45,5)
Percepção de saúde^f^		
Excelente/ótima/muitoboa	903 (30,9)	683 (12,6)
Boa	1.229 (42,0)	3.180 (57,5)
Regular	658 (22,5)	1.519 (25,1)
Pessima/ruim	135 (4,6)	333 (4,9)

^a^ 135 valores faltantes em 2021;

^b^ 1 valor faltante em 2012 e 33 valores faltantes em 2021;

^c^ 2 valores faltantes em 2012 e 171 valores faltantes em 2021;

^d^ 22 valores faltantes em 2012 e 43 valores faltantes em 2021, Associação Brasileira de Estudos Populacionais;

^E^ 4 valores faltantes em 2012 e 2021; e

^f^ 2 valores faltantes em 2021.

As prevalências de utilização dos serviços de saúde nos 30 dias anteriores às pesquisas foram 29,3% (IC95% 27,6; 31,0) em 2012 e 34,4% (IC95% 32,5; 36,3) em 2021, o que representou aumento de 5,1 pontos percentuais. Observou-se aumento significativo de 20,4 pontos percentuais na utilização dos postos de saúde entre 2012 (25,1%; IC95% 21,3; 29,4) e 2021 (45,4%; IC95% 41,8; 49,1). A utilização de consultórios médicos privados diminuiu 17,9 pontos percentuais (43,0; IC95% 38,7; 47,4 em 2012 versus 25,1; IC95% 22,1; 28,4 em 2021), também de forma significativa (Figura 1).

Nos dois anos, mulheres (32,3; IC95% 29,8; 34,7 em 2012 e 38,9; IC95% 36,6; 41,1 em 2021) e indivíduos com percepção de saúde péssima/ruim (61,1%; IC95% 51,5; 70,7 em 2012 e 56,0%; IC95% 50,1; 61,9 em 2021) utilizaram significativamente mais os serviços de saúde, mesmo após ajuste para as variáveis. Também foi observado que, quanto maior a idade, maior a prevalência de utilização em ambos os anos. No entanto, essa associação não permaneceu significativa após ajuste para as demais variáveis (Tabela 2).

Houve associação positiva entre escolaridade e utilização dos serviços de saúde, com maiores prevalências observadas entre aqueles com 12 anos ou mais de escolaridade (35,2; IC95% 30,9; 39,5 em 2012 e 37,7; IC95% 33,6; 41,8 em 2021), estatisticamente significativa apenas em 2012. Os indivíduos da classe D/E apresentaram maiores prevalências de utilização em 2021 (37,9%; IC95% 34,0; 41,7; p-valor 0,010) (Tabela 2).

**Figura 1.** Prevalências ponderadas e intervalos de confiança (IC95%) dos locais de atendimentos entre as pessoas que utilizaram os serviços de saúde. Pelotas, 2012 (n=2.925) e 2021 (n=5.717)

**Tabela 2:** Prevalências ponderadas brutas e ajustadas e intervalos de confiança de 95% (IC95%) de utilização de serviços de saúde de acordo com as características sociodemográficas e de saúde. Pelotas, 2012 (n=2.925) e 2021 (5.717)

Variáveis	Utilização dos serviços de saúde		
	2012		2021		
	Bruta % (IC95%)	Ajustada % (IC95%)	Bruta % (IC95%)	Ajustada % (IC95%)
Sexo	p-valor<0,001	p-valor<0,001	p-valor<0,001	p-valor<0,001
Masculino	22,7 (20,4; 25,2)	23.9 (21,4; 26,6)	28,2 (25,9; 30,6)	29,1 (26,4; 31,8)
Feminino	33,8 (31,4; 36,4)	32,3 (29,8; 34,7)	39,6 (37,2; 42,2)	38,9 (36,6; 41,1)
Faixa etária (anos)	p-valor 0,021^a^	p-valor 0,721^a^	p-valor 0,005^a^	p-valor 0,063^a^
20-29	26,5 (23,0; 30,3)	29,1 (25.0; 33,2)	33,3 (29,3; 37,6)	34,1 (29,6; 38,6)
30-39	25,7 (22,5; 29,2)	27,4 (23,8; 30,9)	31,9 (28,4; 35,6)	32,2 (28,6; 35,8)
40-49	30,0 (26,3; 34,0)	30,3 (26,6; 34,0)	32,1(28,7; 35,6)	32,3 (28,8; 35,9)
50-59	29,8 (25,7; 34,2)	29,1 (25,0; 33,2)	36,0 (32,6; 39,4)	35,7 (32,4; 38,9)
60 ou mais	33,7 (30,0; 37,5)	29,3 (25,5; 33,0)	38,5 (35,8; 41,2)	37,4 (34,8; 40,0)
Raça/cor da pele	p-valor 0,813	p-valor 0,684	p-valor 0,490	p-valor 0,534
Branca	29,0 (27,1; 31,0)	28,7 (26,8; 30,6)	33,8 (31,8; 35,9)	34,1 (32,0; 36,3)
Preta	30,2 (25,5; 35,4)	30,7 (25,6; 35,9)	35,3 (31,3; 39,4)	34,8 (30,9; 38,6)
Parda	29,2 (23,7; 35,3)	29,5 (23,2; 35,9)	38,5 (33,4; 43,9)	38,9 (33,6; 44,2)
Indígena	42,1 (23,6; 63,2)	39,3 (20,8; 57,8)	28,8 (8,2; 64,5)	18,4 (7,5; 54,3)
Amarela	37,5 (17,8; 62,5)	31,2 (11,5; 50,9)	40,6 (14,0; 74,2)	38,3 (5,5; 71,1)
Escolaridade (anos)	p-valor 0,023^a^	p-valor<0,001^a^	p-valor 0,143^a^	p-valor 0,059^a^
Até 4	33,2 (29,6; 37,0)	25,8 (22,5; 29,2)	36,3 (31,7; 41,1)	30,7 (26,1; 35,2)
5-8	26,1 (23,4; 29,0)	25,0 (22,2; 27,8)	36,5 (33,2; 40,0)	34,3 (31,1; 37,4)
9-11	29,9 (26,8; 33,3)	31,3 (27,8; 34,8)	32,7 (30,3; 35,1)	34,4 (31,9; 36,8)
12 ou mais	29,1 (26,0; 32,5)	35,2 (30,9; 39,5)	33,0 (29,9; 36,2)	37,7 (33,6; 41,8)
Classificação econômica (ABEP)^b^	p-valor 0,559^a^	p-valor 0,955^a^	p-valor<0,001^a^	p-valor 0,010^a^
A/B (mais ricos)	28,0 (25,6; 30,7)	28,8 (26,1; 31,4)	30,3 (27,7; 32,9)	31,6 (28,3; 34,9)
C	29,9 (27,3; 32,7)	29,7 (26,9; 32,6)	34,4 (32,3; 36,5)	34,6 (32,5; 36,8)
D/E (mais pobres)	30,3 (25,4; 35,6)	27,7 (22,6; 32,9)	40,0 (36,2; 44,0)	37,9 (34,0; 41,7)
Situação conjugal	p-valor 0,198	p-valor 0,925	p-valor 0,210	p-valor 0,760
Sem companheiro	30,7 (27,9; 33,8)	29,0 (26,3; 31,7)	33,6 (31,4; 35,9)	34,8 (32,3; 37,3)
Com companheiro	28,3 (26,1; 30,5)	29,1 (27,0; 31,3)	35,3 (32,9; 37,8)	34,3 (32,3; 37,3)
Percepção de saúde	p-valor<0,001	p-valor<0,001	p-valor<0,001	p-valor<0,001
Excelente/ótima/muito boa	22,0 (19,4; 24,9)	21,4 (18,6; 24,2)	24,0 (20,4; 28,0)	24,1 (20,1; 28,2)
Boa	25,4 (23,1; 27,8)	25,2 (23,0; 27,4)	31,1 (28,9; 33,4)	31,7 (29,4; 34,1)
Regular	40,2 (36,6; 44,0)	41,3 (37,0; 45,6)	42.4 (39,2; 45.6)	41,4 (38,3; 44,6)
Péssima/ruim	59,2 (50,7; 67,3)	61,1 (51,5; 70,7)	59,3 (53,4; 65,0)	56,0 (50,1; 61,9)

^a^ Teste de tendência.

Associação Brasileira de Estudos Populacionais.

**Figura 2.** Desigualdades absolutas na utilização dos serviços de saúde de acordo com as variáveis de classificação econômica, escolaridade e idade. Pelotas, 2012 (n=2.925) e 2021 (n=5.717)

Em 2012, 54,3% dos atendimentos foram financiados por convênios ou serviços particulares. Entre os que mais utilizaram serviços privados, predominavam mulheres (67,1%), na faixa etária 20-49 anos (57,2%), de raça/cor da pele branca (84,3%), com maior escolaridade (40,3%), com companheiro (59,3%), mais ricos (67,7%) e com percepção de saúde boa (41,3%). Em 2021, 69,8% dos atendimentos foram financiados pelo SUS. Entre os usuários dos serviços públicos, estavam as mulheres (63,2%), com idade entre 20-49 anos (56,7%), da raça/cor da pele branca (72,6%), com 9 a 11 anos de escolaridade (35,4%), com companheiro (52,4%), da classe econômica C (54,1%) e com percepção de saúde boa (50,7%) (Tabela 3).

Em relação às desigualdades absolutas, observaram-se aumento nas diferenças em termos de renda (2012: 0,6; IC95% -6,9; 5,8 e 2021: -5,0; IC95% -1,1; -9,0) e leve redução de acordo com a escolaridade de 2012 (4,9; IC95% 1,5; 11,4) para 2021 (4,4; IC95% 0,7; 8,8), enquanto a diferença relacionada à idade aumentou (2012: -0,7; IC95% -6,4; 5,1 e 2021: 3,1 IC95% -0,8; 6,2), mas não foi significativa em nenhum dos anos. De maneira geral, em 2021, aumentou utilização dos serviços entre os indivíduos mais pobres e mais escolarizados em comparação com 2012 (Figura 2).

As análises de sensibilidade realizadas para 2012 mostraram resultados semelhantes, considerando tanto todos os moradores do domicílio quanto apenas um morador selecionado, com aumento da prevalência de utilização de 29,3% (IC95% 27,6; 31,0) para 30,7% (IC95% 28,2; 33,3). Em relação a 2021, as análises realizadas sem a ponderação do desenho amostral indicaram aumento da prevalência de utilização de 34,4% (IC95% 32,5; 36,3) para 36,5% (IC95% 34,5; 38,4), mantendo padrão similar de desigualdades.

**Tabela 3.** Descrição das características sociodemográficas e de saúde de acordo com o financiamento dos serviços entre as pessoas que utilizaram os serviços de saúde. Pelotas, 2012 (n=856) e 2021 (n=2.085)

Variáveis	Financiamento do serviço			
	2012		2021	
	Público n (%)	Privado n (%)	Público n (%)	Privado n (%)
Sexo				
Masculino	120 (30,7)	153 (32,9)	379 (36,8)	174 (39,3)
Feminino	271 (69,3)	312 (67,1)	1.065 (63,2)	467 (60,7)
Faixa etária (anos)				
20-29	63 (16,1)	99 (21,3)	193 (20,0)	53 (15,3)
30-39	65 (16,6)	74 (15,9)	227 (19,9)	74 (16,2)
40-49	85 (21,7)	93 (20,0)	228 (16,8)	87 (13,6)
50-59	75 (19,2)	78 (16,8)	298 (18,0)	116 (15,5)
60 ou mais	103 (26,3)	121 (26,0)	464 (25,4)	304 (39,4)
Raça/cor da pele				
Branca	287 (73,4)	392 (84,3)	1.052 (72,6)	553 (84,7)
Preta	61 (15,6)	46 (9,9)	262 (18,6)	54 (9,3)
Parda	36 (9,2)	20 (4,3)	112 (8,4)	31 (5,5)
Indígena	5 (1,3)	3 (0,9)	2 (0,1)	0 (0,0)
Amarela	2 (0,5)	4 (0,6)	4 (0,3)	2 (0,4)
Escolaridade (anos)^a^				
Até 4	120 (20,4)	54 (11,6)	65 (15,0)	247 (8,4)
5-8	142 (24,9)	71 (15,3)	112 (33,5)	496 (16,7)
9-11	93 (28,7)	152 (32,8)	203 (35,4)	454 (32,3)
12 ou mais	35 (26,0)	187 (40,3)	249 (16,1)	184 (42,6)
Classificação econômica (ABEP)^b^				
A/B (mais ricos)	81 (21,0)	297 (67,7)	183 (14,5)	249 (39,9)
C	237 (61,6)	140 (30,5)	764 (54,1)	331 (52,8)
D/E (mais pobres)	67 (17,4)	22 (4,8)	490 (31,4)	57 (7,3)
Situação conjugal^c^				
Sem companheiro	176 (45,0)	189 (40,7)	688 (47,6)	282 (44,9)
Com companheiro	215 (55,0)	275 (59,3)	754 (52,4)	359 (55,1)
Percepção de saúde				
Excelente/ótima/muito boa	53 (13,6)	146 (31,4)	108 (7,8)	67 (11,0)
Boa	120 (30,7)	192 (41,3)	693 (50,7)	340 (54,7)
Regular	158 (40,4)	107 (23,0)	489 (32,2)	186 (28,1)
Péssima/ruim	60 (15,4)	20 (4,3)	154 (9,3)	48 (6,2)

^a^ 2 valores faltantes em 2012 e 171 valores faltantes em 2021;

^b^ 22 valores faltantes em 2012 e 43 valores faltantes em 2021, Associação Brasileira de Estudos Populacionais; e

^c^ 4 valores faltantes em 2012 e 2 valores faltantes em 2021.

## Discussão

De 2012 para 2021, a prevalência de utilização de serviços de saúde aumentou de 29,3% para 34,4%. Esse aumento foi marcadamente acentuado na utilização de postos de saúde em 2021 em vez de consultórios médicos em 2012. Por consequência, também houve maior utilização de serviços financiados pelo SUS em 2021. O perfil sociodemográfico nos atendimentos dos serviços públicos e privados nos anos avaliados foram diferentes, contudo houve maior proporção de adultos entre 20 e 49 anos em ambas as situações. Em termos da medida de desigualdade absoluta, aumentaram aquelas relacionadas à renda, e houve leve redução das diferenças por escolaridade. 

Análises dos inquéritos populacionais realizados em São Paulo em 2003, 2008 e 2015 revelaram aumento na demanda, no uso e na resolutividade dos serviços, maior utilização do SUS e mudanças nos padrões de desigualdades educacionais ao longo do tempo ^19^ ^,^ ^20^. Embora realizados em períodos anteriores e em contextos locais distintos, esses resultados foram consistentes com os deste estudo, os quais indicaram ampliação da utilização de serviços públicos por grupos socialmente mais vulneráveis, reforçando a importância do sistema público de saúde na promoção da equidade.

Esse movimento de aumento na utilização do SUS, observado ao longo do tempo, foi abruptamente interrompido com a chegada da pandemia de covid-19, que impactou significativamente a oferta e a demanda por serviços de saúde em todo o país. Análises nacionais anteriores identificaram redução na procura ou na oferta de serviços durante o período pandêmico ^4^ ^-^ ^6^, entretanto os dados aqui apresentados mostraram aumento na utilização entre 2012 e 2021. 

Uma possível explicação para esse resultado está no período de coleta dos dados da pesquisa de 2021, que ocorreu após a fase mais crítica da pandemia. Muitos estudos focaram especificamente em 2020, quando os serviços de saúde estavam mais restritos e a população evitava buscar atendimento por receio de contaminação. Os dados analisados aqui refletiram um momento posterior, marcado pelo início da campanha de vacinação e por maior reorganização dos serviços. O fato de a vacinação contra a covid-19 ter sido implementada prioritariamente no SUS também pode ter estimulado a procura por serviços públicos ^21^. Além disso, os resultados podem indicar uma resposta local mais estruturada do SUS em Pelotas, diante das demandas acumuladas da população e ao agravamento das desigualdades sociais no contexto da pandemia.

A maior utilização dos serviços públicos nesse período também pode ser reflexo de como as desigualdades socioeconômicas, decorrentes do contexto sociocultural e político hostil que afetam os países da América Latina. Os processos históricos de colonização e escravização, que refletem na divisão de recursos até os dias atuais, marcaram e foram exacerbados durante a pandemia de covid-19 ^22^. 

Dois pontos principais podem explicar os resultados deste estudo: o primeiro diz respeito a como a crise pandêmica ampliou a crise econômica no país iniciada em 2016 e promoveu a intensificação do aumento da pobreza no Brasil ^22^ ^,^ ^23^. Por exemplo, as taxas de pobreza aumentaram acentuadamente em 2020 ^23^, e isso também pode ser visualizado a partir deste estudo em que, em geral, houve, de 2012 para 2021, redução no nível socioeconômico da população. No período comparado, houve redução de um pouco mais de 20 pontos percentuais na classe A/B e aumento de cerca de 10 pontos percentuais nas classes C e D/E. 

O segundo ponto diz respeito a como as medidas relacionadas à covid-19, a exemplo do distanciamento social (lockdowns), tiveram impacto sobre os trabalhadores, com cerca de 10 milhões de pessoas deixando a força de trabalho entre o terceiro trimestre de 2019 e o terceiro trimestre de 2020 ^23^. Os dois pontos parecem estar intrinsecamente relacionados. Os efeitos econômicos negativos da pandemia afetaram principalmente as mulheres, os jovens e os menos escolarizados, os quais também tiveram maior probabilidade de perderem seus empregos durante a pandemia ^23^ ^,^ ^24^. 

Neste estudo, foi possível observar como a população mais jovem que em 2012 era usualmente atendida por serviços privados ou serviços pagos passou a ser atendida pelos serviços fornecidos pelo SUS em 2021. Dados sobre a cobertura de planos de saúde em Pelotas, em dezembro de 2012 e 2021, revelaram que a cobertura diminuiu de 16,3% para 15,8% ^25^. Tal resultado sugere a reflexão sobre como o desemprego pode ter afetado essa população, inclusive com possíveis perdas de plano de saúde, pela impossibilidade de pagamento, resultando na necessidade de utilização dos serviços exclusivamente públicos ^23^ ^,^ ^24^. Para a escolaridade, a explicação tende a ser a mesma, embora as desigualdades não tenham sido estatisticamente significativas. 

Este estudo comparou dados de dois períodos distintos, com intervalo de nove anos, inseridos em contextos políticos, econômicos e sanitários diferentes. Essas mudanças, ao longo do tempo, puderam influenciar o padrão de utilização dos serviços de saúde. Além disso, houve diferenças metodológicas importantes entre os estudos, que também limitaram a comparabilidade direta entre os períodos. Em 2012, todos os moradores com 10 anos ou mais do domicílio foram entrevistados, enquanto em 2021 apenas um residente foi selecionado. Embora essa diferença não explique, por si só, o perfil da amostra, observou-se em 2021 a maior proporção de mulheres e pessoas idosas do que o esperado. 

Para mitigar esse possível viés de seleção, foi aplicada uma estratégia de pós-estratificação baseada em projeções populacionais por sexo e idade. Testes de sensibilidade foram realizados para avaliar o impacto dessas diferenças metodológicas e de ponderação, e os resultados se mantiveram consistentes, o que reforça a robustez dos achados principais. Também não foi realizada harmonização dos pesos amostrais entre as coletas, o que pode limitar a comparabilidade direta das medidas absolutas de desigualdade entre os períodos.

Outra limitação foi relacionada à não inclusão de algumas variáveis como a presença de multimorbidade nas análises ajustadas por ser considerada um possível mediador na relação entre fatores sociodemográficos e utilização dos serviços de saúde. A utilização do índice de desigualdade absoluta permitiu uma análise abrangente das desigualdades em saúde, considerando toda a distribuição da variável socioeconômica, e não apenas as categorias extremas. No entanto, como se trata de medida sumarizada, pode mascarar variações relevantes entre categorias intermediárias.

Os achados deste estudo revelaram o aumento da utilização de serviços de saúde, especialmente entre a população mais vulnerável. Embora as desigualdades ainda permaneçam, a maior utilização dos serviços públicos em 2021 refletiu não só uma resposta emergencial às dificuldades econômicas, mas também a importância do SUS em momentos de crise. Isso leva à reflexão sobre a necessidade de fortalecer o sistema de saúde, garantindo que ele seja acessível a todos, especialmente aos grupos mais afetados por desigualdades socioeconômicas.

## Data Availability

Os dados utilizados nesta pesquisa não estão disponíveis em repositórios públicos, mas podem ser acessados mediante solicitação formal. O banco de dados do Consórcio de Mestrado (2012) pode ser solicitado ao Colegiado do Programa de Pós-Graduação em Epidemiologia da Universidade Federal de Pelotas. Os dados do estudo Eai Pelotas (2021) podem ser solicitados por meio de formulário disponível em: https://wp.ufpel.edu.br/eaipelotas/banco-de-dados/. Os questionários estão disponíveis nos respectivos sites: https://guaiaca.ufpel.edu.br/handle/123456789/1952; e https://wp.ufpel.edu.br/eaipelotas/questionario/.
